# Electronic screening, brief intervention, and referral to treatment for poly-drug users in emergency services (the eSBIRTes Project)

**DOI:** 10.1186/1940-0640-8-S1-A20

**Published:** 2013-09-04

**Authors:** Tom F Defillet, Koen Titeca, Karolina Kindt, Bart Rens, Jochen Schrooten, David Möbius

**Affiliations:** 1 Association for Alcohol and other Drug Problems (VAD), Brussels, Belgium; 2 General Hospital Groeninge, Kortrijk, Belgium; 3 General Hospital Sint-Maarten, Duffel, Belgium

## Objective

The general objective of the eSBIRTes project (http://www.esbirtes.eu) was to identify and develop effective tools for screening, brief interventions and referral to treatment for adults presenting at the emergency department (ED) with problems related to poly-drug use. The electronic screening brief intervention and referral to treatment (SBIRT) was pilot tested in four EDs in general hospitals and four first aid stands in dance festivals in Belgium and Hungary.

## Methods

After being treated for their acute health problem, all clients meeting our inclusion criteria were screened using a self-administered modified ASSIST. The screening resulted in 3 possible outcomes: low, moderate, or high risk. Clients whose score was in the low range received an email with a link to local/national drug information website(s). Moderate-risk-clients were referred to an online self help module (DASH) on alcohol, cocaine, GHB, and/or cannabis. Clients in the high-risk range received an overview of treatment centers where they could find professional help; they had also the option to join the self-help module.

## Results

187 attendees completed the screening tool, of whom 119 (63.6%) had a moderate or a high risk for at least one substance. A large proportion of these moderate or high risk users identified themselves as poly drug users, with 30.5% of all respondents receiving a moderate or high risk result for more than one substance. 28% of the participating patients obtained a ‘moderate’ risk score for alcohol, cannabis, cocaine or GHB. They were referred to the online self-help module.

## Conclusion

Within the eSBIRTes project we developed a valuable tool, but the pilot implementation phase has shown that there is a strong need for additional research, especially regarding the setting for this type of intervention. Further research is also needed regarding the conditions under which users of recreational drugs are most open to consider their drug use.

